# First Clinical Application of Aztreonam–Avibactam in Treating Carbapenem-Resistant Enterobacterales: Insights from Therapeutic Drug Monitoring and Pharmacokinetic Simulations

**DOI:** 10.3390/jpm14121135

**Published:** 2024-11-30

**Authors:** Oliver Hölsken, Keno Sponheuer, Franz Weber, Jens Martens-Lobenhoffer, Stefanie M. Bode-Böger, Charlotte Kloft, Sascha Treskatsch, Stefan Angermair

**Affiliations:** 1 Department of Anesthesiology and Intensive Care Medicine, Corporate Member of Freie Universität Berlin and Humboldt Universität zu Berlin, Charité—Universitätsmedizin Berlin, Hindenburgdamm 30, 12203 Berlin, Germany; oliver.hoelsken@charite.de (O.H.); keno.sponheuer@charite.de (K.S.); sascha.treskatsch@charite.de (S.T.); 2 Berlin Institute of Health at Charité—Universitätsmedizin Berlin, BIH Academy, Junior Clinician Scientist Program, Charitéplatz 1, 10117 Berlin, Germany; 3 Institute of Microbiology, Infectious Diseases and Immunology (I-MIDI), Corporate Member of Freie Universität Berlin and HumboldtUniversität zu Berlin, Charité—Universitätsmedizin Berlin, Hindenburgdamm 30, 12203 Berlin, Germany; 4 German Rheumatology Research Center (DRFZ), a Leibnitz Institute, Charitéplatz 1, 10117 Berlin, Germany; 5 Department of Clinical Pharmacy and Biochemistry, Institute of Pharmacy, Freie Universitaet Berlin, Kelchstr. 31, 12169 Berlin, Germany; franz.weber@fu-berlin.de (F.W.); charlotte.kloft@fu-berlin.de (C.K.); 6 Graduate Research Training Program PharMetrX, Freie Universität Berlin/Universität Potsdam, 12169 Berlin, Germany; 7 Department of Clinical Pharmacology, Otto-Von-Guericke University of Magdeburg, University Hospital, Leipziger Strasse 44, 39120 Magdeburg, Germany; jens.martens-lobenhoffer@med.ovgu.de (J.M.-L.); stefanie.bode-boeger@med.ovgu.de (S.M.B.-B.)

**Keywords:** aztreonam, avibactam, carbapenem-resistant enterobacterales, TDM

## Abstract

**Background**: A novel fixed combination of aztreonam (ATM) and avibactam (AVI) offers promising potential to treat infections with carbapenem-resistant *Enterobacterales* (CRE) producing metallo-β-lactamases (MBL). This study aimed to assess the accuracy of population pharmacokinetic (PK) models for ATM-AVI in predicting in vivo concentrations in a critically ill patient with CRE infection during its first clinical use. **Methods**: A 70-year-old male with septic shock due to hospital-acquired pneumonia (HAP) caused by MBL-producing *Klebsiella pneumoniae* was treated with ATM-AVI. Trough and peak serum concentrations (32 samples over 7 days) were measured using liquid chromatography–tandem mass spectrometry (LC-MS/MS). Population PK models were used to simulate complete concentration–time profiles. Bland–Altman analysis assessed model performance by comparing predicted and measured concentrations. **Results**: Median ATM trough concentrations (18.4 mg/L) remained above the minimum inhibitory concentration (MIC) of 1 mg/L for the pathogen. The Bland–Altman analysis demonstrated reasonable agreement between predicted and observed concentrations, with a relative bias (rBias) of −50.5% for ATM and −14.4% for AVI. ATM-AVI ratios remained stable. Clinical improvement and sterile blood cultures within 12 days led to intensive care unit (ICU) discharge. **Conclusions**: Population PK models for ATM-AVI accurately predicted in vivo concentrations in a severely ill patient with HAP. Therapeutic drug monitoring (TDM) with PK modeling ensured optimal antimicrobial exposure and contributed to clinical recovery.

## 1. Introduction

Infections caused by multi-drug-resistant (MDR) gram-negative bacterial pathogens are among the most significant global challenges. Infections by carbapenem-resistant *Enterobacterales* (CRE) are highly concerning due to high morbidity, mortality, and limited treatment options [1,2,3,4]. Especially for OXA-48-like and New Delhi metallo-β-lactamase (NDM)-producing *Klebsiella pneumoniae* infections, recent data support that formerly used antibiotics with in vitro activity like aminoglycosides, colistin, and tigecycline are associated with increased mortality rates and issues of toxicity [5].

Aztreonam (ATM) is the only commercially available monobactam not hydrolyzed by NDM or other metallo-β-lactamases (MBL) enzymes [6]. The combination of ATM with avibactam (AVI), a β-lactamase inhibitor that protects ATM from hydrolysis by inhibition of β-lactamases from Ambler classes A (e.g., ESBLs, KPC), C (AmpC), and D (OXA-48) can be used to eradicate MBL-producing carbapenem-resistant *Enterobacterales* with reduced mortality rates [6,7,8].

On 22 April 2024, the European Medicines Agency (EMA) approved a new fixed combination of ATM-AVI. While the market launch in Germany was planned for September 2024, the ATM-AVI single-product formulation was already available under a compassionate-use program for patients lacking alternative treatment options. However, to our knowledge, no published clinical data regarding therapeutic drug monitoring (TDM) exist to guide treatment in critically ill patients with this novel combination. TDM provides a personalized approach to antimicrobial therapy by measuring drug concentrations in the patient’s plasma, allowing for precise dose adjustments based on individual pharmacokinetic profiles. This is particularly relevant in critically ill patients, as changes in physiology, like cardiovascular disorders, altered fluid balance, increased volume of distribution, and renal and hepatic dysfunction, among others, may potentially lead to inadequate antibiotic exposure, and consequently, treatment failure [9].

ATM acts pharmacodynamically as a time-dependent antibiotic, therefore its antimicrobial activity against specific pathogens is best correlated with the percentage of the dosing interval during which free drug concentrations exceed the minimum inhibitory concentration (MIC), referred to as %*f*T _> MIC_ [10,11,12]. Consequently, antibiotic concentrations above the MIC of the pathogen at the end of the dosing interval indicate effective therapeutic drug levels. In addition to TDM, population-based pharmacokinetic (PK) models can be employed to anticipate the consequences of a dosing regimen even before the commencement of antibiotic therapy. This enables dose adjustments based on plasma concentrations before a steady state has been reached [13].

The objective of this study was to ascertain the accuracy of existing population PK models for ATM-AVI to predict the in vivo concentrations in a severely ill patient with hospital-acquired pneumonia (HAP) caused by MBL-producing *Klebsiella pneumoniae*. To test this hypothesis, we retrospectively assessed TDM data and compared serum concentrations with predicted levels. This is the first clinical use of the novel single-product formulation of ATM-AVI following its approval, providing a real-world example of its application in a highly resistant infection. We focus on the critical role of TDM and the potential of PK modeling and simulation approaches to pursue optimal antibiotic exposure.

## 2. Materials and Methods

### 2.1. Patient Characteristics

A 70-year-old male patient with a severe pulmonary infection and septic shock (Acute Physiology and Chronic Health Evaluation version II (APACHE II) score 19) caused by CRE was treated in the interdisciplinary intensive care unit (ICU) of the Department of Anesthesiology and Intensive Care Medicine at the Charité—Universitätsmedizin Berlin, Campus Benjamin Franklin. The patient had a complex medical history, including heart failure with mildly reduced ejection fraction, obstructive sleep apnea, diabetes mellitus, and morbid obesity, with a body mass index (BMI) of 45 kg/m^2^ (151 kg, 185 cm). Patient demographics are summarized in Table 1. The patient had undergone multiple surgical procedures and experienced complications following a periprosthetic femoral fracture, resulting in a prolonged hospitalization period that commenced three months before ICU admission. Two months following his last surgery, the patient developed pneumogenic sepsis. Microbiological cultures identified *Klebsiella pneumoniae*, resistant to carbapenems due to OXA-48 and NDM production, isolated from urine, blood cultures, and bronchoalveolar lavage fluid. The patient exhibited numerous risk factors for MDR infections, including previous antibiotic use due to a wound infection and a recent prolonged hospitalization period [14].

The study consisted of a retrospective analysis of clinical data obtained through standardized operating procedures for therapeutic drug monitoring (TDM). The study was conducted in line with the Declaration of Helsinki. Ethical approval was granted by the Institutional Review Board (IRB) of the Charité—Universitätsmedizin Berlin (approval number EA/239/19, approval date: 7 February 2020).

### 2.2. Antimicrobial Sensibility Testing

Antimicrobial susceptibility testing (AST) was performed according to EUCAST standards. For routine AST, the Vitek 2™ system (bioMérieux, Nuertingen, Germany) was used. For extended testing of antibiotics for MDR *Enterobacterales*, we used Micronaut™ S MDR MIC/GN2 plates (Bruker Daltonics, Bremen, Germany), including Aztreonam/Avibactam with a fixed Avibactam concentration at 4 mg/L. Automated reading of Micronaut ™ plates was performed using a Tecan Sunrise™ Reader (Tecan, Männedorf, Switzerland) and MCN6 Release 125 software. AST demonstrated that all isolates were sensitive to ATM-AVI, with a MIC of ≤1 mg/L, well below the PK/PD (pharmacodynamic) breakpoint of ≤4 mg/L for *Enterobacterales* (Supplementary Figure S1) [15].

### 2.3. Therapeutic Drug Monitoring Methodology and Data Collection

Blood samples for TDM were collected daily for a period of 7 days, starting on the first day following the commencement of ATM-AVI. Both trough (pre-dose) and peak concentrations of ATM and AVI were measured daily. The plasma samples were subsequently frozen at −80 °C and analyzed in a retrospective manner.

ATM and AVI were simultaneously quantified in human plasma by LC-MS/MS analogously to the method described previously for ceftazidime and AVI [16]. In short, 20 µL plasma was mixed with 20 µL internal standard solution (each 20 mg/L D_6_-ATM and ^13^C_5_-AVI in water). Proteins were precipitated by the addition of 60 µL methanol. After centrifugation, 20 µL of the clear supernatant was diluted by 200 µL buffer solution (1 g ammonium formate and 1 mL formic acid in 1 L water, pH 3.5). Then, 20 µL of this mixture was injected into the HPLC system. The analytes were separated on a ReproSil-Pur 120 C18-AQ column with the dimensions 150 × 3 mm by gradient elution, starting with a composition of 95% of the described buffer solution and 5% methanol, evolving to 45% buffer solution and 55% methanol after 5 min. The flow rate was 400 µL/min. Retention times were 3.6 min for AVI and 8.4 min for ATM. The mass spectrometer worked in the negative ESI mode and recorded the fragment ions, m/z 434 → 96 and 440 → 96 for ATM and its internal standard and m/z 264 → 96 and 269 → 96 for AVI and its internal standard, respectively. Calibration functions were linear in the range of 2–100 mg/L for ATM and 0.5–25 mg/L for AVI, with inter-day relative standard deviations of 3.6% for ATM and 3.9% for AVI at the lower end of the calibration ranges. The TDM measurements were used to determine ATM-AVI ratios over time.

### 2.4. Pharmacokinetic Models and Simulations

For both ATM and AVI, PK models were provided by Pfizer Pharma, Berlin, Germany [17], based on data from relevant patient populations. The ATM population PK model was a two-compartment model with zero-order infusion and first-order elimination based on 107 healthy subjects and subjects with renal impairment. Inter-individual variability (IIV) and inter-occasion variability (IOV) were included with exponential models for clearance (CL), the central volume of distribution (V1), and the peripheral volume of distribution (V2). Covariate effects included creatinine clearance (CLCR) on clearance using a two-part linear function, with a steeper slope for CLCR values below 80 mL/min. Age was included as a covariate on V1 for patients older than 65. The nonlinear protein binding of ATM was modeled using a maximum binding capacity (Bmax) and a concentration at half-maximal binding (B50). Theory-based allometric scaling total body weight (TBW) was applied, with exponents of 1 for volumes (V1, V2) and 0.75 for CL and inter-compartmental clearance (Q).

The provided AVI population PK model was a two-compartment model with zero-order infusion and first-order elimination based on 1836 healthy subjects, subjects with renal impairment, and patients with cIAI (complicated intra-abdominal infection) or cUTI (complicated urinary tract infection) from the ceftazidime-AVI development program. IIV was included with exponential models for CL, Q, V1, and V2. Covariate effects included CLCR on CL, with CL decreasing proportionally for CLCR < 80 mL/min and increasing linearly for CLCR ≥ 80 mL/min. Furthermore, end-stage renal disease (ESRD) and augmented renal clearance (ARC), dialysis status, and APACHE II score were included as categorical covariates on CL. The central volume of distribution was adjusted for TBW in the different subpopulations and ARC groups.

Both models were utilized to simulate total drug concentrations resulting from the dosing regimen applied to the underlying patient. Stochastic simulations (n = 1000) applying IIV and IOV for ATM and IIV for AVI were performed with the final estimates of both models reported [17]. The simulations were conducted utilizing the R package ‘mrgsolve’ (v1.0.9) [18].

Due to the severe obesity (Table 1) of the patient and the predominately renal elimination [19] of both compounds in both models, adjusted body weight (ABW) based on the ideal body weight (IBW) and the TBW were used instead of TBW within the allometric scaling and the estimated CLCR formula based on Cockcroft-Gault [20] for both compounds (Equations (1)–(3)). IBW was 79.5 kg (Equation (1)) and ABW was 108.1 kg (Equation (2)).

(1) $$IBW\;[kg]=\left\{\begin{array}{c} \;50+0.9055\cdot \left(height\;\left[cm\right]-152.4\right);\;if\;male\\ \;45.5+0.9055\cdot \left(height\;\left[cm\right]-152.4\right);\;iffemale \end{array}\right.$$

(2) $$ABW[kg]=IBW\;\left[kg\right]+0.4\cdot \left(TBW\;\left[kg\right]-IBW\;\left[kg\right]\right)$$

(3) $${CLCR}_{CG\text{\_}ABW}[mL/min]=\left\{\begin{array}{c} \;\frac{\left(140-age\;[years]\right)\cdot ABW\;[kg]\;}{72\cdot creatinine\;[mg/dL]};\;if\;male\\ \frac{\left(140-age\;[years]\right)\cdot ABW\;[kg]\cdot 0.85}{72\cdot creatinine\;[mg/dL]};if\;female \end{array}\right.$$

### 2.5. Statistical Analysis

Exploratory data analysis was conducted to evaluate the PK profiles of ATM and AVI. The Bland–Altman analysis [21] was used to compare the median predicted and measured concentrations of ATM and AVI at two different time points: 30 min before the next dose and 30 min after the start of infusion. Bias was calculated as the mean difference between predicted and observed values, and the limits of agreement were defined as bias ± 1.96 times the standard deviation of the differences. Additionally, the relative bias (rBias) and relative root mean square error (rRMSE) were calculated to quantify the accuracy and variability of the predictions for each drug.

## 3. Results

Following the microbiological findings and susceptibility testing, which revealed carbapenem-resistant *Klebsiella pneumoniae* with sensitivity to ATM-AVI (Supplementary Figure S1), the initial empiric anti-infective therapy was specifically adjusted to ATM-AVI under compassionate use. For the first five days, the patient received a regimen of 1.5 g ATM and 0.5 g AVI every 8 h (*q*8h), administered intravenously over 3 h. Following clinical improvement and increased renal excretion, on the fifth day, the dosing frequency was increased to every 6 h (*q*6h). No drug-related adverse events, such as anemia, diarrhea, or elevated liver enzymes, were observed during the 10-day treatment period. Table 2 provides a summary of the clinical and laboratory parameters associated with the treatment. The patient was successfully extubated, transferred back to the geriatric ward, and discharged home 54 days later.

### 3.1. Therapeutic Drug Monitoring Results

**Figure 1:**
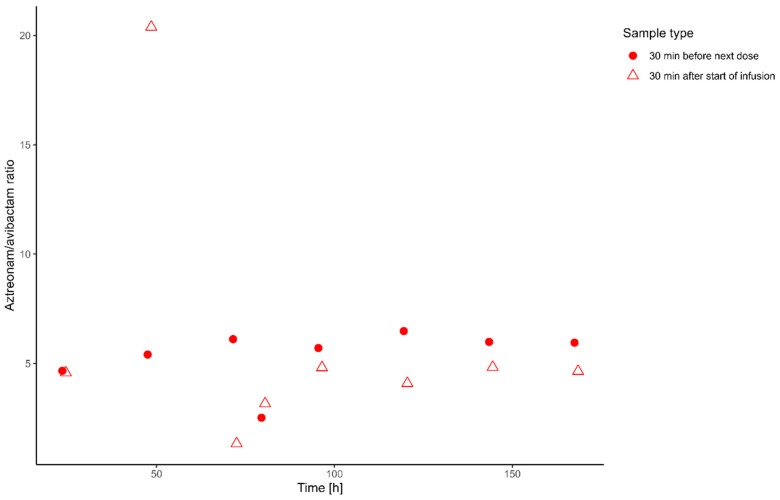
Ratio of drug concentrations for aztreonam (ATM) and avibactam (AVI) over the time course of treatment. Filled circles represent the ratio of Therapeutic Drug Monitoring (TDM) sample concentrations of ATM and AVI taken 30 min before the next dose, while triangles indicate the ratio of concentrations measured 30 min after the start of infusion.

In total, 32 (16 ATM, 16 AVI) samples were analyzed over 7 days. TDM data showed that the median ATM concentration 30 min before the next dose was 18.4 mg/L (min: 7.88 mg/L, max: 24.6 mg/L) and, thereby, consistently remained above the MIC of 1 mg/L. The median ATM concentration 30 min after the start of infusion was 32.95 mg/L (min: 17.16 mg/L, max: 61.42 mg/L). For AVI, median concentrations were 3.08 mg/L (min: 1.95 mg/L, max: 4.75 mg/L) 30 min before and 7.05 mg/L (min: 4.06 mg/L, max: 12.1 mg/L) 30 min after the start of infusion. The ATM/AVI ratio demonstrated greater stability for the samples collected 30 min before the subsequent dose administration (Figure 1). For those samples, a median ratio of 5.82 (range: 2.53–6.48) was identified. For the samples taken 30 min after the start of the infusion, higher variability was identified, with a median ratio of 4.61 (range: 1.35–20.4). Representative chromatograms demonstrating the quantification of AVI and ATM are shown in Supplementary Figure S2.

### 3.2. Pharmacokinetic Simulations

**Figure 2:**
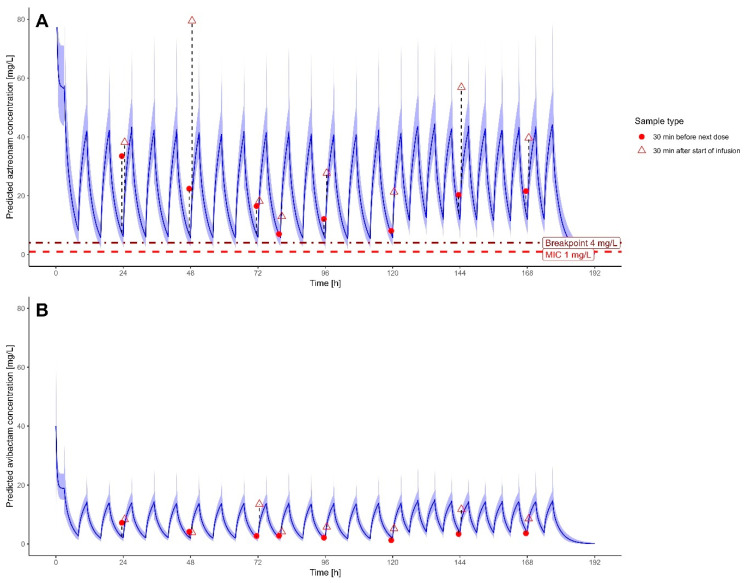
Simulated aztreonam (ATM) (**A**) and avibactam (AVI) (**B**) concentrations over the time course of treatment. Blue lines indicated the median predicted concentration-time profile based on stochastic simulations (n = 1000) of patients with the same patient characteristics as the investigated patient, including interindividual variability (**A**,**B**) and inter-occasion variability (**A**). Blue ribbons represent the 90% prediction interval (5th–95th percentile). Filled circles indicate measured concentrations 30 min before the next dose and triangles indicate samples 30 min after the start of infusion. The red dashed horizontal line in A represents the exemplary MIC of 1 mg/L based on the antibiogram and the dark red dash-dotted line shows the clinical breakpoint of 4 mg/L. Black dashed vertical lines indicate the difference between measured and median predicted concentrations. MIC: Minimum inhibitory concentration.

**Figure 3:**
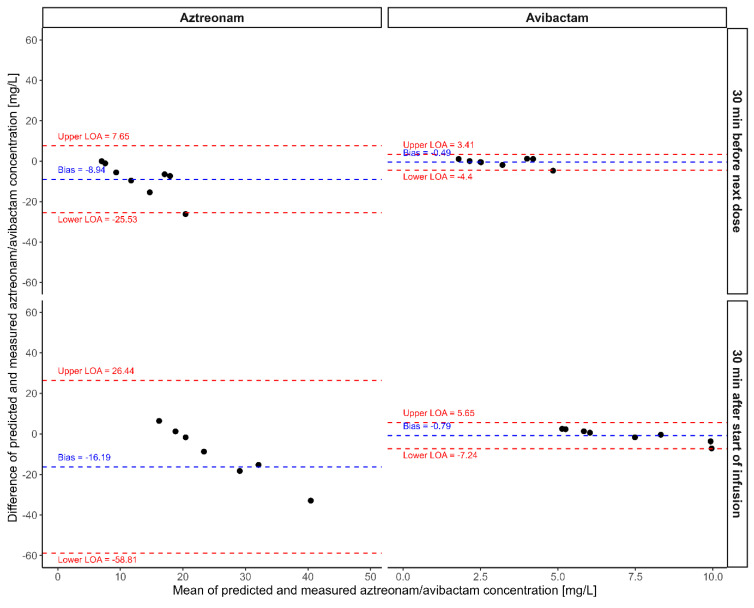
Bland–Altman plots comparing the median predicted and measured concentrations of aztreonam (ATM) and avibactam (AVI) at two different time points: 30 min before the next dose (top row) and 30 min after the start of infusion (bottom row). The plots on the left display ATM, while those on the right display AVI concentrations. The difference between the median predicted concentration and the measured concentration is plotted against their mean. The dashed blue line represents the bias (mean difference) and the dashed red lines represent the upper and lower limits of agreement (LOA), calculated as bias ± 1.96 × standard deviation of the differences.

Both PK models were successfully utilized to simulate the total concentrations of ATM and AVI throughout the antibiotic therapy (i.e., 3-h infusions of 1.5 g ATM and 0.5 g AVI after a bolus of the same amounts; 12–16 June 2024 *q*8h, 17–19 June 2024 *q*6h). Overall, both models described the TDM data well, although the discrepancies were higher for the ATM samples taken 30 min after the start of infusion (Figure 2). All simulated ATM minimum concentrations were above the MIC of 1 mg/L throughout the time course, and the medians of the predicted ATM concentrations were also above the breakpoint throughout the entire time course of treatment. In Figure 3, Bland–Altman analysis revealed biases (Equation (4)) between median predicted and measured TDM concentrations for ATM of −8.94 mg/L (30 min before the next dose) and −16.2 mg/L (30 min after the start of infusion). For AVI, the biases were −0.49 mg/L and −0.79 mg/L at the same respective time points.

The relative bias (rBias) and relative root mean square error (rRMSE) were calculated for ATM and AVI at the two different sampling time points (Equations (5) and (6)).

(4) $$BIAS=\frac{1}{N}\;\sum_{1}^{i}\left({predicted}_{i}-{observed}_{i}\right)$$

(5) $$rBIAS=\frac{1}{N}\;\sum_{1}^{i}\left(\frac{{predicted}_{i}-{observed}_{i}}{{observed}_{i}}\right)\times 100$$

(6) $$rRMSE=\sqrt{\frac{1}{N}\;\sum_{1}^{i}\left(\frac{{\left({predicted}_{i}-{observed}_{i}\right)}^{2}}{{\left({observed}_{i}\right)}^{2}}\right)}\times 100$$

For ATM, the rBias was −50.5% and −44.0% 30 min before the next dose and 30 min after the start of the infusion, respectively. The corresponding rRMSE values were 67.5% and 70.6%, indicating a substantial negative bias and variability between predicted and measured concentrations. In contrast, AVI showed lower rBias values of −14.4% (30 min before the next dose) and −10.4% (30 min after the start of infusion), with rRMSE values of 56.7% and 41.6%, respectively. These results suggest that the predicted concentrations for AVI were closer to the measured values and had less variability compared to ATM.

The elimination rate constants (k_e_) based on the patient’s characteristics and the utilized PK models were estimated to be 0.277 h^−1^ for ATM and 0.384 h^−1^ for AVI. The half-life values based on the patient’s characteristics and the utilized PK models were estimated to be 2.51 h for ATM and 1.80 h for AVI, aligning well with recent studies for both ATM [22] and AVI [23]. The steady-state average concentrations for the studied patient, based on the PK model used, were estimated to be 22.4 mg/L for ATM and 4.97 mg/L for AVI during a *q*8h dosing regime. For a *q*6h dosing regime, the estimated concentrations were 29.8 mg/L and 6.63 mg/L, respectively.

## 4. Discussion

The fixed combination of ATM-AVI is a recently approved treatment option for infections caused by gram-negative bacteria, including MBL-producing pathogens. It is approved for the treatment of complicated intra-abdominal infection, HAP (including ventilator-associated pneumonia—VAP), and complicated urinary tract infections (including pyelonephritis) in adult patients with limited therapeutic options. As demonstrated in the phase III study REVISIT, it is comparable to meropenem in treating severe infections caused by gram-negative bacteria [24]. EUCAST has set the clinical MIC breakpoint for ATM-AVI in *Enterobacterales* at ≤4 mg/L for susceptible strains, with resistance defined at >4 mg/L [15].

To the best of our knowledge, this is the first clinical use of ATM-AVI following Phase III trials worldwide incorporating TDM measurements. In this study, the concentration–time profiles for ATM and AVI, generated from stochastic simulations based on two previously published pharmacokinetic models by Pfizer^®^ [17], demonstrated a high degree of alignment with the measured concentrations. Antibiotic concentrations, taken 30 min before the next dose and 30 min post-infusion, were predominantly within the 90% prediction interval, thereby indicating that the population pharmacokinetic models accurately reflected the drug exposure in this patient. Because of the considerable increase in concentrations that occur following the infusion of ATM-AVI, even minor discrepancies in the timing of the 30-min post-infusion TDM measurement can result in pronounced fluctuations compared to the measurements taken before the next dose. This can explain the larger biases between the median predicted and measured concentrations in the post-infusion peak measurements for both components. The use of ABW to calculate the CLCR_CG_, a requisite covariate in both models, markedly enhanced the predictive capacity of both models concerning the actual TDM data. Future studies should further investigate the effect of obesity status on ATM and AVI exposure and relevant covariates such as CLCR_CG_ on drug clearances, especially in cases where models were developed using non-obese data.

One of the main challenges in treating our patient was their extreme obesity (adiposity WHO III). In comparison to the existing clinical data from the phase 3 trial [24], the studied patient’s TBW (151 kg) and BMI (45 kg/m^2^) exceeded the maximum weight described for the patients in the HAP/VAP study arm. However, only 7 out of the 177 patients exhibited infections caused by MBL-positive bacteria. Of these, only two (28.6%) demonstrated a clinical response classified as “cure”, while three (42.9%) exhibited a “failure” and two (28.6%) an “indeterminate” response. Five patients with HAP exhibited infections due to carbapenem-resistant Klebsiella pneumoniae, with NDM as the underlying resistance mechanism in four cases (Supplementary Table S1). These patients had comparable demographic characteristics, including a mean age of approximately 70 years, and a high prevalence of acute and chronic comorbidities. They also share proven risk factors for carbapenem-resistant *Klebsiella pneumoniae* infection, including ICU admission and central venous catheterization, mechanical ventilation, surgery, and prior or concurrent antibiotic exposure [25]. Furthermore, the majority exhibited a resistant pattern to ATM monotherapy; however, the supplementation of AVI resulted in a treatable MIC value of 2 mg/L or below.

The APACHE II score of 19 is notable for this patient, particularly when considered in the context of the overall study population. However, it is comparable with the subgroup of patients treated for HAP (mean 16.5, range 10–30). Additionally, the patient exhibited an intact estimated glomerular filtration rate (eGFR) of > 90 mL/min, septic shock, and an extensive cardiovascular comorbidities profile. All described factors have been identified as risk factors for target non-attainment of beta-lactam antibiotics due to the challenging predictability of PK/PD in critically ill patients [26]. Loading doses of ATM-AVI followed by maintenance infusions four times daily has been shown to optimize the probability of target attainment, especially in the initial period [17] compared to the not pharmacodynamically optimized dosing regimen of ceftazidime–avibactam and ATM as recommended by the Infectious Diseases Society of America (IDSA) [7]. The simultaneous infusion of ATM-AVI with likely synchronous concentration profiles at the site of infection and the avoidance of a redundant beta-lactam (ceftazidime) support the use of this single-formulation product even though it has not directly been tested against ceftazidime–avibactam and ATM in clinical trials.

To optimize drug delivery, a prolonged infusion of three hours was selected by international guidelines [27]. It is noteworthy that the simulated ATM concentrations remained consistently above the MIC of 1 mg/L, thereby ensuring approximately 100% *f*T _> MIC_ and sustaining efficacy against the identified *Klebsiella pneumoniae* strain. Furthermore, the median concentrations exceeded the clinical breakpoint of 4 mg/L, thus confirming the appropriateness of the selected dosing regimen. For AVI, serum levels should be above the critical threshold concentration of 2.5 mg/L for at least 50% of the therapeutic time [28].

Although beta-lactam antibiotics are generally well tolerated, there are reports about ATM-induced encephalopathy [29] and liver enzyme elevations [30]. Therefore, it seems reasonable to also avoid unnecessarily high serum levels of ATM. Throughout the course of treatment, the ATM/AVI ratio remained consistent, indicating that in clinical practice, isolated ATM monitoring may be sufficient. This relationship would be analogous to that observed between ceftazidime and AVI [16]. Further TDM data from a larger cohort of critically ill patients are required to confirm this finding.

After prospective validation with more real-world clinical data, both utilized PK models have the potential to be integrated into easy-to-use model-informed precision dosing (MIPD) instruments [13,31]. MIPD instruments can enable healthcare professionals to decide on dosing adjustments directly at the patient’s bedside by considering relevant patient, pathogen, and TDM data. This is of high relevance because routine TDM measurements of ATM and AVI are not yet available even in a university hospital setting and dose adjustments need to be applied as soon as possible to correct levels that are out of the target range.

The principal limitation of this study is the small sample size of a single patient, which limits the extent to which the findings can be generalized. Further TDM data from critically ill patients treated with a single formulation of ATM-AVI are required to corroborate these findings. For reasons of cost-effectiveness and rational use of antibiotics, ATM-AVI will continue to be used as a reserve antibiotic combination for the treatment of patients with MBL-producing strains. The data from the REVISIT trial underscore the low incidence of infections with MBL-positive strains, with only ten isolates testing positive for MBL in a population of 422 patients recruited over 5 years across 20 countries [24]. It is therefore our contention that the data yielded by this trial will prove invaluable in the absence of data from larger cohorts. To address this concern, we are currently planning a multi-center prospective observational trial with the potential to generate additional data on PK modeling and TDM in critically ill patients in the future.

Our study highlights the importance of incorporating TDM alongside pharmacokinetic modeling and simulation methods to optimize dosing and maintain drug exposure within the therapeutic window, particularly in critically ill patients with complex infections. Inadequate dosing not only compromises clinical efficacy but also increases the risk of promoting antimicrobial resistance.

## 5. Conclusions

TDM-guided ATM-AVI proved to be an effective therapeutic option for treating a critically ill patient with a complicated infection caused by multidrug-resistant *Klebsiella pneumoniae*. The clinically complex patient required dosing adjustments; however, they benefited from these modifications and prolonged infusion strategies. TDM combined with pharmacokinetic simulations were key to optimizing dosing and ensuring that drug concentrations remained within the therapeutic range. This case emphasizes the value of TDM in tailoring treatments for complex cases and highlights the role of ATM-AVI as a promising option for patients with limited therapeutic choices.

## Figures and Tables

**Table 1:** Baseline patient demographics upon ICU admission.

Age in years	70
Sex	Male
Height, cm	185
Weight, kg	151
Body Mass Index (BMI), kg/m^2^	45 (WHO Class III—Morbid Obesity)
APACHE II Score	19
Primary diagnosis	Hospital-acquired pneumonia (HAP), pneumogenic sepsis
Causative pathogen	Klebsiella pneumoniae (NDM and OXA-48 carbapenemase-producing strain)
Co-morbidities	- Heart failure (mildly reduced ejection fraction) - Obstructive sleep apnea - Diabetes mellitus
Hospitalization	ICU stay following a prolonged hospital course due to surgical complications

Abbreviations: NDM: New Delhi Metallo-β-lactamase. OXA-48: Oxacillinase-48. APACHE II Score: Acute Physiology and Chronic Health Evaluation version II Score.

**Table 2:** Patient clinical course, intensive care scores, and laboratory values.

	Day 0 (ICU Admission)	Day 1	Day 3	Day 7	ICU Discharge	Hospital Discharge (Day 66)
**Intensive Care Scores**						
SOFA	6	7	5	4	-	-
Max. Norepinephrine (µg/kg/min)	0.1	0.06	0.02	0	0	-
Highest lactate level (mg/dL)	16	15	15	8	7	-
**Laboratory Values**						
WBC (/nL)	19.91	11.37	4.92	4.68	8.02	11.12
Thrombocytes (/nL)	215	217	214	212	230	164
eGFR (mL/min)	93	87	88	97	93	73
CLCR**_CG_ABW_** (mL/min)	143.6	123.6	126.5	158.6		
Creatinine (mg/dL)	0.74	0.86	0.84	0.67	0.74	1.03
Urea (mg/dL)	14	19	31	20	12	32
Plasma Albumin (g/dL)	-	3.2	-	-	3.4	-
Transaminases						
ALT (U/L)	23	13	8	10	16	<5
AST (U/L)	22	36	28	15	18	19
Bilirubin (mg/dL)	0.66	0.77	0.34	0.32	0.33	0.57
INR	1.4	1.51	1.21	1.26	1.30	1.2

Abbreviations: ABW, Adjusted Body Weight; ALT, Alanine Aminotransferase; AST, Aspartate Aminotransferase; CLCR_CG_ABW_, Creatinine Clearance (Cockcroft-Gault) based on adjusted body weight; eGFR, Estimated Glomerular Filtration Rate using CKD-EPI (Chronic Kidney Disease Epidemiology Collaboration); ICU, Intensive Care Unit; INR, International Normalized Ratio; max, maximum; WBC, White Blood Cells, SOFA, Sequential Organ Failure Assessment.

## Data Availability

The TDM data and analysis code used in this study are available from the corresponding author upon reasonable request.

## Supplementary Materials

The following supporting information can be downloaded at: https://www.mdpi.com/article/10.3390/jpm14121135/s1, Figure S1: Antibiotic susceptibility of the *K. pneumoniae* isolate recovered from our patient; Figure S2: Representative chromatograms of avibactam and aztreonam in patient blood samples; Table S1: Comparison of our patient to patients with pneumonia caused by MBL-producing *K. pneumoniae*, adapted from the REVISIT-Trial.

## Abbreviations

The following abbreviations are used in this manuscript:

AST	Antimicrobial susceptibility testing
ATM	Aztreonam
AVI	Avibactam
BAL	Bronchoalveolar Lavage
BMI	Body Mass Index
CL	Clearance
CLCR	Creatinine Clearance
CLSI	Clinical and Laboratory Standards Institute
cIAI	Complicated Intra-abdominal Infection
cUTI	Complicated Urinary Tract Infection
eGFR	Estimated Glomerular Filtration Rate
EUCAST	European Committee on Antimicrobial Susceptibility Testing
ESBL	Extended-spectrum Beta-lactamase
ESRD	End-stage Renal Disease
IIV	Inter-individual Variability
IOV	Inter-occasion Variability
k_e_	Elimination rate constant
LC-MS/MS	Liquid Chromatography–tandem Mass Spectrometry
MIC	Minimal Inhibitory Concentration
NDM	New Delhi Metallo-β-lactamase
OXA-48	Oxacillinase-48
PK/PD	Pharmacokinetic/Pharmacodynamic
rBias	Relative bias
rRMSE	Relative root mean square error
SOFA	Sequential Organ Failure Assessment
TBW	Total Body Weight
TDM	Therapeutic Drug Monitoring
WBC	White Blood Cells
WHO	World Health Organization
HAP	Hospital-acquired Pneumonia
CRE	Carbapenem-resistant Enterobacterales
KPC	Klebsiella pneumoniae Carbapenemase
