# Supplementary material for: First Clinical Application of Aztreonam–Avibactam in Treating Carbapenem-Resistant Enterobacterales: Insights from Therapeutic Drug Monitoring and Pharmacokinetic Simulations
Source: J Pers Med. 2024 Nov 30;14(12):1135. doi: 10.3390/jpm14121135 (PMC11676034; doi:10.3390/jpm14121135)
Supplement: Supplementary file 1 [file jpm-14-01135-s001.zip › jpm-3307438-supplementary.pdf]

**Supplementary Figure S1.** Antibiotic susceptibility of the *K. pneumoniae* isolate recovered from our patient; Interpretation per CLSI criteria for all antibiotics; Abbreviations: BAL: bronchoalveolar lavage; R, resistant; S, susceptible; I, intermediate; MIC: minimal inhibition concentration; CLSI, Clinical and Laboratory Standards Institute: For MIC determination, the concentration of AVI is fixed at 4 mg/L.

| **Antibiotic** | MIC, mg/L | | |
|---|---|---|---|
| | Urine | BAL | Blood |
| Ampicillin | R >=32.0 | R≥32.0 | R >=32.0 |
| Ampicillin/Sulbactam | - | R≥32.0 | R >=32.0 |
| Piperacillin | R | R≥128.0 | R >=128 |
| Piperacillin/Tazobactam | R >=128 | R 64.0 | R >=128 |
| Cefuroxime | R >=64.0 | R≥64.0 | R >=64.0 |
| Ceftazidime | R >=64.0 | R≥64.0 | R >=64.0 |
| Cefepime | R 32.0 | R 32.0 | R >=64.0 |
| Ceftazidime/Avibactam | R >=64.0 | R≥64.0 | R >=64.0 |
| Ceftozolane/Tazobactam | R >=64.0 | R≥64.0 | R >=64.0 |
| Cefiderocol | R | S | R |
| Ertapenem | R >=8.0 | R≥8.0 | R >=8.0 |
| Imipenem | R 2.0 | R≥16.0 | R 8.0 |
| Meropenem | R >=16.0 | R≥16.0 | R 8.0 |
| Aztreonam | R >=128 | R≥128.0 | R >=128 |
| Aztreonam/Avibactam | . <=1.0 | <= 1.0[1] | . <=1.0 |
| Ciprofloxacin | R >=4.0 | R≥4.0 | R >=4.0 |
| Levofloxacin | - | R | R |
| Amikacin | R >=64.0 | R≥64.0 | R >=64.0 |
| Gentamicin | - | S 2.0 | S <=1.0 |
| Tobramycin | R >=32.0 | R≥32.0 | R >=32.0 |
| Minocycline | . 4.0 | . 4.0 | . >=8.0 |
| Colistin | S 0.5 | S 1.0 | S 0.5 |
| Fosfomycin | R 48.0 | S <= 16 | R 64.0 |
| Cotrimoxazole | R >=320 | R ≥ 320 | R >=320 |

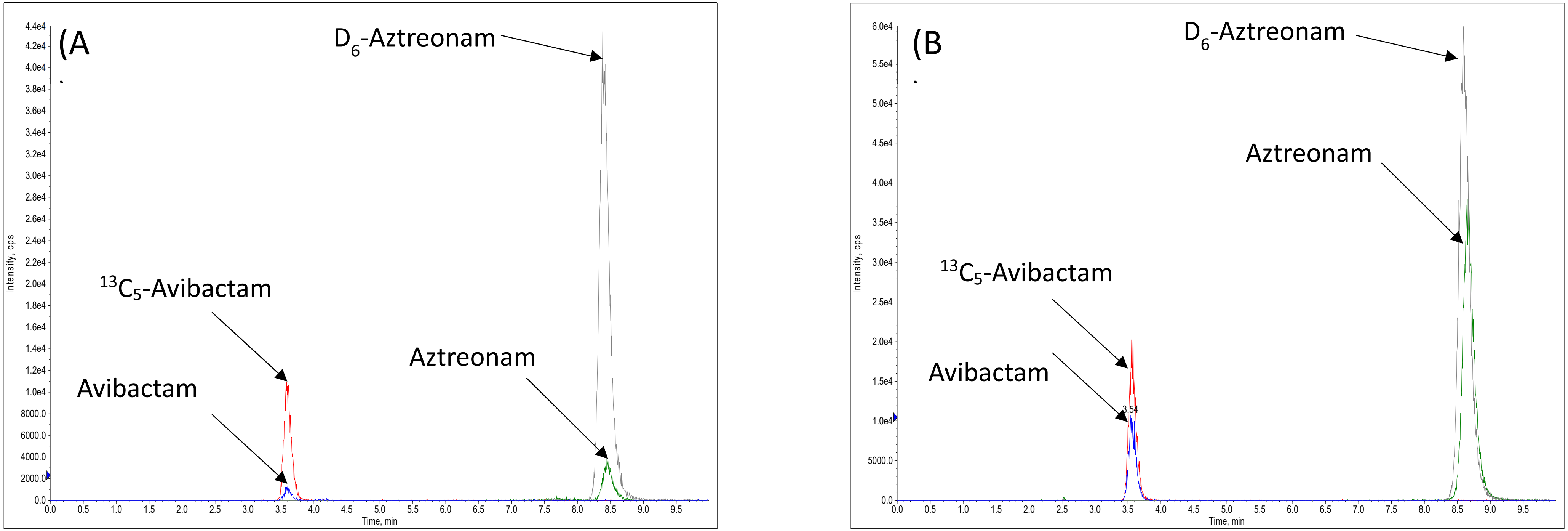


**Supplementary Figure S2**. Representative chromatograms of avibactam and aztreonam in patient blood samples. Panel (A): calibration level 1 containing 0.5 μg/mL avibactam and 2 μg/mL aztreonam. Panel (B): patient sample containing 2.70 μg/mL avibactam and 16.5 μg/mL aztreonam.

**Supplementary Table S1.** Comparison of our patient to patients with pneumonia caused by MBL-producing *K. pneumoniae*, adapted from the REVISIT-Trial [24]. Abbreviations: ATM: Aztreonam, AVI: Avibactam; HAP: hospital-acquired pneumonia; *K. pneumoniae*: *Klebsiella pneumoniae*; MBL: metallo-β-lactamase; Minimal inhibition concentration; N/A: not applicable; NDM: New Delhi metallo-β-lactamase; VAP: ventilator-associated pneumonia; VIM: Verona Integron-encoded metallo-β-lactamase.

*According to clinical trial definition; Mean age of REVISIT patients: 71.8 years, Range: 68-68 years, Standard deviation: 4.2 years

| | Our patient | REVISIT 1 | REVISIT 2 | REVISIT 6 | REVISIT 7 | REVISIT 8 |
|---|---|---|---|---|---|---|
| Age (years) | 70 | 75 | 73 | 76 | 67 | 68 |
| Gender | Male | Male | Female | Female | Female | Male |
| Infection | Monomicrobial HAP | Monomicrobial VAP | Monomicrobial VAP | Monomicrobial HAP | Monomicrobial HAP | Polymicrobial VAP |
| Co-morbidities | Obstructive spleep apnea, obesity, congestive heart failure (mildly reduced ejection fraction) | Pneumothorax, atrial fibrillation, myocardial infarction, hypokalaemia, sacral ulcer, acute kidney injury, encephalopathy, Parkinson's disease, hypoalbuminemia and malnutrition. Considered a previous treatment failure after undergoing treatment with ciprofloxacin, cefepime and vancomycin | Atrial fibrillation, left bundle branch block, hypertension, congestive heart failure, diabetes mellitus, anaemia, hypoalbuminemia, leg amputation, and traumatic subarachnoid haemorrhage. Considered a previous treatment failure after undergoing treatment with piperacillin-tazobactam, tigecycline and daptomycin. | Acute stroke, ischaemic heart disease, arterial hypertension, atrial fibrillation, type 2 diabetes, COVID-19 infection, and sacral, gluteal and perineal bedsores. | Laparoscopic cholecystectomy, drainage of choledochus, complete external biliary fistula, and endoscopic insertion of an enteral feeding tube into the duodenum. | Laryngeal cancer, chemotherapy, local actinotherapy, laryngectomy, tracheostomy, aortic aneurysm, and community-acquired pneumonia. |
| Treatment Group | N/A | ATM+AVI for 14 days | ATM+AVI+Vancoymcin for 7days | ATM+AVI for 1 day | ATM+AVI for 14 days | ATM+AVI for 10 days |
| Pathogen | *K. pneumoniae* | *K. pneumoniae* | *K. pneumoniae* | *K. pneumoniae* | *K. pneumoniae* | *K. pneumoniae* |
| MBL Subtype | NDM | NDM | VIM | NDM | NDM | NDM |
| ATM MIC | > 128 | 0.25 | 64 | 64 | 64 | 64 |
| ATM AVI MIC | ≤ 1 | 0.25 | 2 | 0.5 | 0.5 | 0.5 |
| Clinical response* | N/A | Failure | Failure | Indeterminate | Cure | Cure |
| 28-day-survival | Alive | Alive | Alive | Dead | Alive | Alive |
